# Age-Patterns of Malaria Vary with Severity, Transmission Intensity and Seasonality in Sub-Saharan Africa: A Systematic Review and Pooled Analysis

**DOI:** 10.1371/journal.pone.0008988

**Published:** 2010-02-01

**Authors:** Ilona Carneiro, Arantxa Roca-Feltrer, Jamie T. Griffin, Lucy Smith, Marcel Tanner, Joanna Armstrong Schellenberg, Brian Greenwood, David Schellenberg

**Affiliations:** 1 Disease Control and Vector Biology Unit, Department of Infectious and Tropical Diseases, London School of Hygiene and Tropical Medicine, London, United Kingdom; 2 Department of Infectious Disease Epidemiology, MRC Centre for Outbreak Analysis and Modelling, Imperial College London, London, United Kingdom; 3 Swiss Tropical Institute, Basel, Switzerland; The Kenya Medical Research Institute, Kenya

## Abstract

**Background:**

There is evidence that the age-pattern of *Plasmodium falciparum* malaria varies with transmission intensity. A better understanding of how this varies with the severity of outcome and across a range of transmission settings could enable locally appropriate targeting of interventions to those most at risk. We have, therefore, undertaken a pooled analysis of existing data from multiple sites to enable a comprehensive overview of the age-patterns of malaria outcomes under different epidemiological conditions in sub-Saharan Africa.

**Methodology/Principal Findings:**

A systematic review using PubMed and CAB Abstracts (1980–2005), contacts with experts and searching bibliographies identified epidemiological studies with data on the age distribution of children with *P. falciparum* clinical malaria, hospital admissions with malaria and malaria-diagnosed mortality. Studies were allocated to a 3×2 matrix of intensity and seasonality of malaria transmission. Maximum likelihood methods were used to fit five continuous probability distributions to the percentage of each outcome by age for each of the six transmission scenarios. The best-fitting distributions are presented graphically, together with the estimated median age for each outcome. Clinical malaria incidence was relatively evenly distributed across the first 10 years of life for all transmission scenarios. Hospital admissions with malaria were more concentrated in younger children, with this effect being even more pronounced for malaria-diagnosed deaths. For all outcomes, the burden of malaria shifted towards younger ages with increasing transmission intensity, although marked seasonality moderated this effect.

**Conclusions:**

The most severe consequences of *P. falciparum* malaria were concentrated in the youngest age groups across all settings. Despite recently observed declines in malaria transmission in several countries, which will shift the burden of malaria cases towards older children, it is still appropriate to target strategies for preventing malaria mortality and severe morbidity at very young children who will continue to bear the brunt of malaria deaths in Sub-Saharan Africa.

## Introduction

The peak prevalence of *Plasmodium falciparum* malaria infection is known to shift to younger age groups as transmission intensity increases [1]; there is ample evidence for this from the field [2]. However, while there is a general acceptance that the same shift in peak age occurs for severe malaria [3]–[8], there has been limited evidence of this for uncomplicated clinical malaria [9], [10]. A recent analysis of data from seven demographic surveillance sites in sub-Saharan Africa suggested that the peak age of malaria mortality is similar across sites, despite differences in transmission intensity [11].

The “peak shift” phenomenon has been described for malaria and other infections to which immunity is acquired [12], [13] and has two implications for control. First, identification of the age groups bearing the greatest burden of clinical malaria for a given transmission setting would enable interventions to be targeted to those worst affected. Secondly, it implies that reported declines in malaria transmission intensity, and future progress towards malaria elimination, will result in a shift of malaria morbidity towards older children, as has recently been observed [14]–[16].

Several research groups have undertaken exercises to quantify the burden of malaria regionally or globally [17]–[21], or to assess how the intensity of malaria of different severities varies with transmission intensity [22]–[24]. However, there has been no comprehensive assessment of the comparative age-patterns of different malaria outcomes across a wide range of epidemiological settings. This study builds on previous data abstraction efforts to collate and analyse the age distribution among children of clinical malaria, hospital admissions with malaria and deaths diagnosed as malaria. Data are abstracted from epidemiological studies across sub-Saharan Africa. We have not considered data from outside sub-Saharan Africa because of the very different epidemiology of malaria in many of these areas with P. vivax playing an important role. We use statistical modelling of these data to describe the age-patterns of *P. falciparum* malaria for six broad epidemiological settings categorised according to distinct combinations of malaria transmission intensity and seasonality.

## Methods

### Data Sources and Definitions

**Figure 1:**
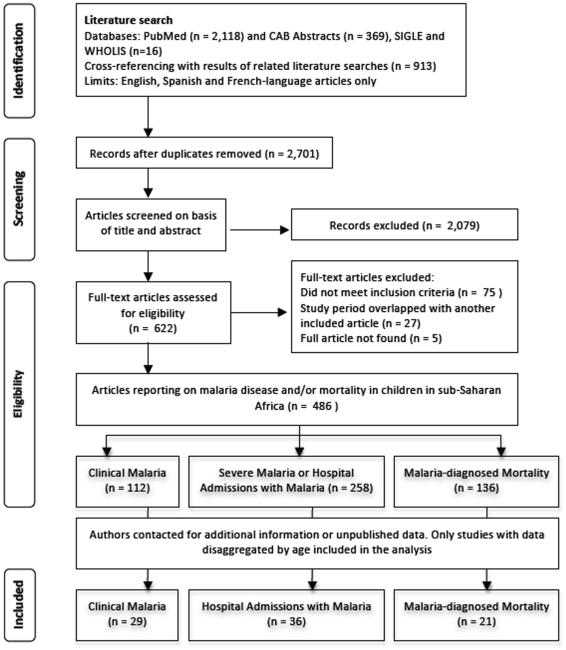
Systematic literature review process. Flow diagram describing the systematic review of literature on the age-breakdown of uncomplicated clinical malaria, hospital admissions with malaria and malaria-diagnosed deaths.

Systematic literature reviews were undertaken using the PubMed and CAB Abstracts (BIDS) online abstracting databases, the WHO publication Library (WHOLIS: www.who.int/library/databases/en/) and the SIGLE grey literature database (www.opensigle.inist.fr), conducting key author searches and cross-checking the bibliographies of references identified and of previous literature reviews on malaria morbidity and anaemia [25]. A review of studies that measured entomological inoculation rates (EIR) between 1970–2005 was undertaken in November 2005 using combinations of the following search terms: EIR, entomologic* inoculation rate, sporozoites inoculation rate, anoph*, vector* capacity, biting rate, sporozoite rate, sporozoites index, malaria transmission, entomol*, malaria control, light trap, pyreth* spray, human bait. The EIR studies were screened for quality, including sampling methods, length of sampling period and frequency of sampling. A review of studies that measured parasite prevalence between 1980–2006 was undertaken in February 2006 using combinations of the following search terms: malaria, falciparum, parasit*, epidemiology, parasitology, transmission, prevalence, morbidity, cross-sectional survey, child, infant. A review of studies that measured malaria morbidity and mortality outcomes between 1980–2005 was undertaken in October 2005 (see Figure 1) using combinations of the following search terms: malaria, falciparum, morbidity, mortality, epidemiology. An additional review of studies on all-cause hospital admissions from 1980–2008 was undertaken in July 2008 using combinations of the following search terms: hospitalization, hospitals/statistics and numerical data, hospitals/trends, hospitals/utilization, inpatients, hospital mortality, pediatrics. Only studies undertaken from 1980 in sub-Saharan Africa in areas endemic for *Plasmodium falciparum* and which reported on malaria outcomes in children (under 15 years of age) were included. Age of clinical malaria episodes and malaria-diagnosed deaths was obtained from community-based studies of a representative sample of the population, while age of hospital admissions with malaria was identified from hospital-based studies. Only data from baseline periods or control arms of intervention studies were included, to prevent confounding of the relationships under investigation, especially by transmission reducing interventions.

All relevant references were collected, examined and key information abstracted directly into a relational database form in Microsoft Access, which was developed to link the studies using geographical co-ordinates. When additional information was needed, authors were contacted for further details. A number of authors shared individual-level datasets, or a more detailed age-breakdown of malaria outcome data than was publically available.

Data on the incidence of clinical malaria were obtained from longitudinal studies using either active or passive case detection. Case definitions varied according to (i) whether or not reported history of fever was considered, and over what time period (24 or 48 hours), (ii) whether a concurrent axillary (≥37.5°C) or rectal (≥38.5°C) temperature measurement was considered, and (iii) whether a parasite density threshold was used. An age-specific fever density threshold calculated according to previously described methods to ascertain the malaria-attributable fraction of fevers in endemic areas [26] was used in some studies [27]–[32]. Where data were available to us [33]–[40], the number of malaria-attributable fevers in each age group was calculated using the following formula:
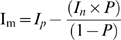
where I*_m_* is the incidence of fevers truly attributable to malaria, *I_p_* is the incidence of fevers associated with malaria parasites, *I_n_* is the incidence of fevers not associated with malaria parasites and P is the prevalence of *P. falciparum*. In some cases, data on age-specific fever incidence or parasite prevalence was obtained from other studies from the same site and linearly interpolated if the same age-categories were not available.

It was not possible to estimate the malaria-attributable incidence of fevers adjusting for changes in a parasite density threshold with age for 11 of the 29 studies included in this analysis. Of these, 6/11 used a fixed fever density threshold, with 3/6 calculating a locally specific density threshold based on the malaria-attributable fraction. A sensitivity analysis was undertaken to assess the impact of using different threshold densities on the results.

Hospital admissions with malaria were all (or, in some cases, a proportional sample) of admissions to the paediatric ward of a given hospital who had a positive rapid diagnostic test or blood flim for malaria. All-cause hospital admissions were all admissions to a paediatric ward. Malaria-diagnosed deaths were obtained from studies using recognised verbal autopsy methods, and include all acute febrile illness deaths minus those with an obvious alternative cause. Data on all-cause deaths were obtained from the malaria-diagnosed deaths literature review, and were not searched for separately.

### Classification of Study Site Transmission Intensity and Seasonality

Given the imprecision and temporal variability of EIR estimates, malaria transmission intensity was arbitrarily categorised into three broad categories as low (EIR<10 bites per person per year (pppy)), medium (EIR 10–100 bites pppy) or high (EIR>100 bites pppy). Settings where malaria is epidemic were not included. Few studies reported good quality EIR estimates, and the previously described log-linear relationship between EIR and parasite prevalence [41], [42] was independently confirmed from the studies identified through our searches. The log-linear relationship identified cut-offs of <25%, 25–60% and >60% parasite prevalence in children <5 years of age to be approximately concordant with the low, medium and high transmission intensity using EIR [25]. Using the geo-referenced relational database, each study was assigned to a transmission intensity category on the basis of EIR or parasite prevalence studies from within the same second administrative level, matched as closely as possible in time.

The seasonality of malaria transmission for a given setting was difficult to define, with contradictory descriptions sometimes given by different authors for the same site. Preliminary analysis of monthly incidence data revealed a continuum of seasonal patterns, which could be arbitrarily divided into those with ≥75% of episodes concentrated in ≤6 months of the year, considered to have “marked seasonality”, and those with no marked seasonality [43]. The MARA (Mapping Malaria in Africa) maps of seasonality of climate suitability for malaria were consulted [44] if consistent definitions were not available from published references. In a few instances where no additional data on transmission intensity or seasonality were available for a given study, malaria experts with local knowledge were consulted. See Tables S1, S2, and S3 for criteria and sources used to allocate each study to one of six cells of a transmission intensity-seasonality matrix as presented in Table 1: low, medium, high transmission, with marked or no marked seasonality.

**Table 1:** Distribution of research study countries and sites by matrix of transmission intensity and seasonality.

Outcome	Seasonality	Transmission Intensity (Entomological Inoculation Rate [bites per person per year (pppy)])		
		<10 bites pppy*	10–100 bites pppy	>100 bites pppy
**Clinical malaria**	**Marked seasonality**	Mali (Sotuba), Senegal (Central & South Dakar)	Cameroon (Ebolakounou), Mali (Sotuba), Mozambique (Maputo), Senegal (Niakhar), The Gambia (Upper Baddibu)	Burkina Faso (Nouna), Ghana (Kassena-Nankhana), Mali (Doneguebougou)
	**No marked seasonality**	Ghana (Prampram), Uganda (Kampala),	Gabon (Lambarene), Mozambique (Manhica), Tanzania (Ifakara, Muheza highlands)	Benin (Atlantic Coast), Cameroon (Koundou), Cote d'Ivoire (Korhogoro), Ghana (Afigya Sekyere), Kenya (Asembo Bay), Senegal (Dielmo), Tanzania (Idete, Muheza, Tanga)
**Hospital admissions with malaria parasites**	**Marked Seasonality**	Guinea Bissau (Bissau), Senegal (Dakar), Sudan (Geradif), Tanzania (Huruma, Kibosho), The Gambia (Banjul, Sibanor)	Burkina Faso (Ougadougou), Mozambique (Maputo), Tanzania (Same), Zambia (Choma)	Burkina Faso (Sorou & Nayala)
	**No marked seasonality**	-	Benin (Cotonou), D. R. Congo (Brazzaville, Kinshasa), Gabon (Lambarene, Libreville), Ghana (Kumasi), Kenya (Kilifi), Malawi (Blantyre, Mangochi, Zomba), Mozambique (Manhica), Nigeria (Ilorin), Tanzania (Ifakara, Moshi)	Ghana (Tamale), Tanzania (Tanga)
**Malaria-diagnosed mortality**	**Marked Seasonality**	-	The Gambia (Farafenni, South Bank, Upper River Division), Senegal (Niakhar)	Burkina Faso (Kourweogo, Nouna, Oubritenga), Ghana (Kassena-Nankhana), Senegal (Bandafassi)
	**No marked seasonality**	-	Kenya (Kilifi), Mozambique (Manhica), Tanzania (Ifakara, Rufiji)	Burundi (Nyanzalac), D. R. Congo (Katana), Guinea (Mandiana), Kenya (Asembo Bay, Kisumu), Tanzania (Bagamoyo)

### Statistical Analyses

The malaria outcomes described are incidence of clinical malaria with fever, numbers of hospital admissions with malaria parasites and numbers of malaria-diagnosed deaths in the community. Data on age of all-cause hospital admissions and all-cause mortality were also abstracted for comparative purposes. Data on each outcome were abstracted by age (in months under five-years of age, if possible, and then by year) up to 15 years. Studies based on very small sample sizes (<50 cases or person-years at risk) were excluded because the percentage age-distributions could easily be spurious. For the hospital admissions with malaria and malaria-diagnosed deaths, 1 [45] and 2 [46], [47] studies respectively which reported only on children less than 5 years of age were excluded as these would otherwise skew the age distribution as there was no denominator for analysis of these outcomes.

All data were analysed using Stata 10.1 (StataCorp. 2007. Stata Statistical Software: Release 10. College Station, TX: StataCorp LP). A user-written (JG) command (details available on request from the authors) was used to censor the data at different age intervals (interval-censored) and to use data with different maximum age values (right-truncated). This enabled the inclusion of studies that reported on different age ranges and used different age groupings. The percentage of each outcome by age (0–10 years - excluding neonates for malaria-diagnosed mortality) was calculated for each study. Studies from the same research setting were included if there was no temporal overlap. However, it was not possible to adjust the analyses for intra-cluster correlation within site due to small numbers of studies from the same site. Data from sites in the same cell of the transmission matrix were analysed together and five continuous probability distributions (gamma, Weibull, log-normal, log-logistic, exponential) were fitted to the data using a time-to-event likelihood for severe events, or Poisson likelihood to account for the person-time at risk for the incidence of clinical malaria. The distributions with the lowest AIC (Akaike Information Criterion) value were identified as the best fitting [48]. For the analysis of malaria-diagnosed deaths, the fitted distributions were re-scaled to exclude children under 1 month of age, as neonatal deaths are difficult to attribute and malaria is not considered a major problem in this age group. The median age was calculated as the 50^th^ percentile of the best-fitting distribution for each outcome and transmission scenario.

## Results

A total of 29, 36 and 21 studies were included in the analysis of clinical malaria, hospital admissions with malaria, and malaria-diagnosed mortality respectively (see Table 2). In addition, 22 studies on all-cause admissions and 28 studies on all-cause mortality were also analysed (data not shown). For malaria and all-cause hospital admissions, there were no data from areas with no marked seasonality and low transmission intensity. For all-cause admissions there were also no data for areas of high intensity and marked seasonality. However, the analyses included 3 studies from areas with sporadic or no malaria transmission. For malaria-diagnosed mortality, there were no data from areas with low transmission.

**Table 2:** Number of studies, (research sites) and [sources of data] included in the analyses, with best-fitting probability distributions.

Outcome	Seasonality	Transmission Intensity (Entomological Inoculation Rate [bites per person per year (pppy)])		
		<10 bites pppy	10–100 bites pppy	>100 bites pppy
**Clinical malaria**	**Marked seasonality**	3 (2) [10], [39], [60] Log-normal	5 (5) [10], [29], [32], [61], [62] Log-normal	4 (3) [10], [37], [45], [63] Log-normal
	**No marked seasonality**	2 (2) [27], [38] Log-normal	6 (5) [34], [36], [40], [64]–[66] Gamma	9 (9) [28]–[31], [33], [35], [36], [67]–[69] Log-normal
**Hospital admissions with malaria parasites**	**Marked Seasonality**	9 (6) [6], [70]–[76] Log-logistic	6 (4) [5], [6], [77]–[80] Log-logistic	1 (1) [5] Log-logistic
	**No marked seasonality**	0-	18 (14) [6], [14], [74], [81]–[90] Log-normal	2 (2) [6], [91] Log-logistic
**Malaria-diagnosed mortality**	**Marked Seasonality**	0-	6 (4) [92]–[97] Log-normal	6 (5) [11], [96], [98], [99] Log-logistic
	**No marked seasonality**	0-	4 (4) [11], [100] Log-logistic	5 (5) [11], [101]–[104] Gamma

**Figure 2:**
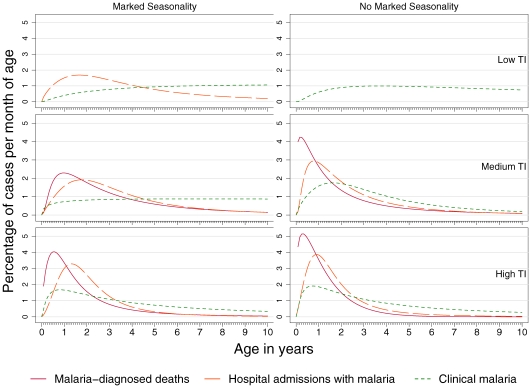
Age-patterns of *P. falciparum* malaria in Sub-Saharan Africa. Percentage of uncomplicated clinical malaria, hospital admissions with malaria and malaria-diagnosed deaths per month of age in children under ten years of age, by transmission intensity (TI) and seasonality of malaria transmission.

Figure 2 shows the percentage of each outcome by age for children under 10 years, such that the integral of the curve is equal to one, i.e. 100% of expected cases. Clinical malaria is relatively evenly distributed across all ages with a shift towards younger age groups as transmission intensity increases, both in areas of non-marked and marked seasonality. The median age for clinical malaria ranges from 32 months (Inter-quartile range (IQR): 15, 61) in settings of highly intense and not markedly seasonal transmission, to 72 months (IQR: 45, 97) in settings of low intensity and markedly seasonal transmission. In six studies, the estimation of malaria-attributable fevers resulted in negative values for some age-groups, and these were considered to be zero cases of malaria-attributable fevers. For one study [37], all age-groups had a higher asymptomatic prevalence of infection than prevalence of fevers associated with parasites, resulting in this study contributing an incidence of zero malaria-attributable cases across the age-groups 6–24 months.

A sensitivity analysis using no threshold density and a fixed density cut-off for those studies for which data were available suggested that the age-pattern would be slightly more evenly distributed across age groups if an age-specific cut-off was not used (data not shown). Therefore, the results presented here are likely to partially underestimate the proportion of clinical malaria that occurs in younger age groups, especially for areas of marked seasonality where 7/12 studies used a fixed density threshold or no threshold density to define clinical malaria.

Hospital admissions with malaria parasites are more concentrated in younger children than is clinical malaria in all settings, and these severe cases become more concentrated in younger ages with increasing transmission intensity and less seasonality. The median age ranges from 17 months (IQR: 10, 29) at high, not markedly seasonal transmission to 36 months (IQR: 20, 60) at low and markedly seasonal transmission.

Malaria-diagnosed mortality is more focussed in younger children than admissions with malaria in all settings for which there are comparative data. Once more there is a distinctive shift of the peak age towards infants as transmission becomes more intense. The median age for malaria-diagnosed mortality ranges from 12 months (IQR: 6, 22) at high, not markedly seasonal transmission to 28 months (IQR: 15, 51) at medium and markedly seasonal transmission.

For all three outcomes, the age-patterns are less biased towards younger ages in areas of marked seasonality than in areas without marked seasonality for a given intensity of transmission. Table 3 shows the median ages and inter-quartile ranges for each outcome for each cell of the transmission matrix.

**Table 3:** Median ages in months (inter-quartile range) for each outcome for each transmission matrix cell.

Outcome	Seasonality	Transmission Intensity (Entomological Inoculation Rate [bites per person per year (pppy)])		
		<10 bites pppy	10–100 bites pppy	>100 bites pppy
**Clinical malaria**	**Marked seasonality**	72 (45, 97)	65 (35, 92)	37 (17, 67)
	**No marked seasonality**	62 (37, 89)	37 (20, 60)	33 (16, 62)
**Hospital admissions with malaria parasites**	**Marked Seasonality**	36 (20, 60)	34 (20, 55)	22 (14, 34)
	**No marked seasonality**	-	22 (12, 41)	17 (10, 29)
**Malaria-diagnosed mortality**	**Marked Seasonality**	-	28 (15, 51)	15 (8, 29)
	**No marked seasonality**	-	17 (7, 36)	12 (6, 22)

## Discussion

We have compiled and analysed available data on *P. falciparum* malaria outcomes of different severities across all transmission intensities in sub-Saharan Africa. Our analyses confirm previous findings that the peak age of uncomplicated clinical malaria declines with increasing transmission intensity. The most striking finding, however, was the extent to which hospital admissions with malaria, and especially malaria-diagnosed deaths, occur in the youngest age groups regardless of transmission intensity. This has implications for the age-targeting of malaria control strategies.

For uncomplicated clinical malaria, the distribution is relatively even across all ages under-10 years with a slight tendency for the age-pattern to shift towards younger age groups with increasing transmission intensity. This is similar, although less pronounced, to previously published comparative results from Senegal [9] and Mali [10], some of which was excluded from this pooled analysis (due to lack of denominator data for the Ndiop study [9]).

It is likely that inclusion of data without an age-specific density threshold for one third of the studies will have resulted in a dampening of the peak-shift effect reported here. The incidence of non-malarial fever with incidental parasitaemia would be expected to increase with age as parasite tolerance is acquired. The use of no fever density threshold would therefore result in an overestimate of the proportion of cases in older age groups, while the use of a fixed “average” density threshold would result in an underestimate of the proportion of cases at younger ages and an overestimate of cases at older ages. Estimation of the *true* malaria-attributable incidence for many studies was hampered by a lack of site-specific information on either all-cause fever or parasite prevalence for the same age-ranges covered by our data. An increase in the specificity of the case definition would shift the age-distribution of malaria-attributable fevers further towards younger ages, and this would increase with transmission intensity, as parasite tolerance would develop earlier in life. In addition, not all studies removed the time at risk after an antimalarial treatment, and this will serve to underestimate the true incidence. This bias will be more pronounced in the peak incidence age-groups resulting in a dampening of the age-patterns, especially at higher transmission intensities.

Hospital admissions for malaria are concentrated in children under-5 years of age in all settings, with a shift towards younger ages with increasing intensity of malaria transmission. This is consistent with previous reports that have compared data across sites with different transmission intensities [3], [4], as well as within sites with observed declines in transmission intensity over time [14]–[16]. The age-distributions of different syndromes of severe malarial disease have previously been shown to vary with transmission intensity [23], [49], [50]. A comprehensive analysis of specific outcomes across a range of transmission settings has been undertaken and will be presented elsewhere (Roca-Feltrer *et al.*, manuscript in preparation).

Our analysis shows that, across all ranges of transmission intensity, malaria-diagnosed mortality is focussed in the very young. This is in keeping with recently published data on age-specific malaria mortality rates from a number of sites in Africa, which showed the peak age of malaria deaths to be in children under 1 year of age [11].

Our findings have implications for malaria control programmes that aim to tackle the worst effects of malaria. Based on the age-pattern of clinical episodes and severe disease, the burden of malaria deaths in the very young would likely be underestimated. The situation is compounded by the fact that, in sub-Saharan Africa, the majority of malaria deaths occur outside hospital and are therefore less visible than cases of severe disease. Age-targeted strategies such as intermittent preventive treatment of infants (IPTi) [51] and children (IPTc) [52], and vaccination against malaria [53] are currently being investigated and are likely to have a role to play in settings with moderate and high transmission [54]. The intensity of malaria is expected to decline in many areas as efforts are made towards elimination [55], which will require a move towards universal coverage with preventive interventions [56]. However, it is likely that many of the countries in Africa will not reach this situation for many years to come.

The age-patterns of all-cause mortality and all-cause hospital admissions did not vary by malaria transmission setting (data not shown). This may be due, in part, to the strong influence of neonatal disease and death on these age-patterns, which we were unable to deconstruct, as most data sets did not sufficiently disaggregate the neonatal data. One exception was for all-cause admissions at medium transmission intensity and marked seasonality of malaria, for which the data were only available for a smaller age-range (no neonates for 2 studies and <8 years for all 3 studies).

The effect of seasonality of malaria transmission on the three outcomes was to dampen the impact of transmission intensity on the age-patterns. Within each intensity category, the peak age is lower for settings with no marked seasonality than for those with marked seasonality. This has not previously been reported, but could have been predicted. The “peak-shift” phenomenon is consistent with gradually acquired protective immunity [12], and the effect of seasonal transmission would be to reduce the cumulative exposure of individuals to malaria and hence their acquisition of immunity.

The fact that the peak age declines with increasing severity of the outcome, implies that protection against the more severe forms of malaria is acquired more rapidly than protection against clinical malaria, as previously suggested [57]. This phenomenon may be compounded by a tendency for those who are unable to develop a protective immune response to succumb to the disease and die in their early years. The strong bias for severe malaria outcomes to be concentrated in infants across different transmission intensities lends weight to the suggestion that protection against severe falciparum malaria may be acquired with age, independently of, or in synergy with prior exposure through mechanisms that are not yet fully understood.

As with any major data abstraction exercise our analyses had some limitations. The data abstracted showed a bias towards established research settings, with gaps in the availability of data from countries in central Africa. The lack of data from settings with no marked seasonality and low intensity probably reflects a lack of research facilities in such settings; most malaria research sites are in areas of medium to high transmission intensity. Nevertheless, data from research settings are likely to be susceptible to the Hawthorne effect (where the presence of a dedicated research team alters the disease epidemiology) as a result of increased implementation of interventions potentially leading to a decline in transmission [14]–[16]. With further progress in malaria control, there is likely to be an increase in data for low transmission intensity settings. These geographic and epidemiological ‘black holes’ in malaria research need to be filled urgently.

In addition to the lack of data from some epidemiological categories, it was generally difficult to obtain age-specific data on malaria morbidity and mortality, which are usually not published in sufficient detail to be included in a thorough statistical analysis. This was ameliorated to some extent by contacting authors. Allocating sites to transmission intensity bands may also have been subject to some misclassification, both in terms of a lack of local data on intensity parameters (EIR or parasite prevalence), and also in relation to temporal changes. It is very rare to have geographically and temporally matched data on EIR or parasite prevalence and morbidity/mortality outcomes, and local or expert knowledge was sought where possible to support the classification. The categorisation of settings as having marked seasonality or no marked seasonality may also be subject to some disagreement, as seasonality is a continuum, with most settings showing some seasonal peaks and some perennial settings having a distinct clustering of cases. However, sensitivity analyses of the definition used suggested that sites showed consistent patterns of seasonality for different malaria outcomes and across several years [43]. Given the limitations in defining the epidemiological context for a particular setting, the analyses and results were restricted to six broad categories defined by the transmission matrix.

The allocation of some sites may change over time, e.g. the measured EIR for Sotuba, Mali was 12 in 1999 and 4 in 2000 [10]. We temporally matched the outcome data to the epidemiological data, where possible, so that sites may appear in more than one cell of the matrix depending on the timing of the studies. The relationships presented here are between the burden of disease, and the intensity and seasonality of malaria transmission, and as such, are independent of the changing epidemiology of malaria.

We attempted to estimate the incidence of truly malaria-attributable fevers, rather than fevers incidentally associated with parasites, by using the age-specific all-cause fever incidence and parasite prevalence, where this was available. In addition to a lack of suitable data to enable this adjustment to be made for many studies, it is likely that the use of non-contemporary data reduced the utility of this approach. For example, using cross-sectional parasite prevalence from peak transmission seasons to adjust the incidence over a longer period of time could result in an underestimate of malaria-attributable cases.

It is also important to note the limitations in diagnosing malaria deaths through the verbal autopsy method, which suffers from a lack of a distinct symptom complex for malaria [11], [58]. In addition, malaria is also likely to be an underlying cause in many deaths, but missed as the final diagnosis if a more distinct terminal event occurs. While it is difficult to estimate the incidence of true malaria-attributable mortality, it is likely that the mis-classification of malaria deaths will be internally consistent within a study, and thus analysis of percentage age-distributions will suffer less from such errors than would cross-site comparisons.

No attempt was made here to assess the relationship between the *incidence* (as opposed to the percentage distribution) of disease and transmission intensity using these data. Such exercises have previously been undertaken [4], [22]–[24], but there are several limitations to such an endeavour, limiting the number of studies that can be included. While most recent estimates of mortality come from community-based demographic surveillance systems, it was difficult to obtain age-specific denominator population data from many of the older studies. Data on severe malaria outcomes can only be obtained at the hospital level, as continued community surveillance will reduce the progression to severe disease. Unfortunately, it is difficult to estimate hospital catchment populations, as individuals may not access the nearest facility, and local access issues (geography, socio-economics, treatment-seeking behaviour) make it difficult to develop a standardised definition. While mathematical modelling may go some way to alleviating this issue [59] it is reliant on additional data from the site, which is not always available. Estimates of incidence are thus hampered by the lack of a denominator population for many studies of severe outcomes. In addition, given that most data come from established research settings, the presence of a dedicated malaria research team, with improved health infrastructure and/or surveillance, is itself likely to reduce the burden of malaria that would otherwise occur in that setting. In addition, local practices and access to interventions and health care may confound the relationship between transmission intensity and the age-specific burden of malaria. Nevertheless, this relationship should be the focus of any future extensions to such an exercise, as this will be most informative for public health planning.

The data presented here show that the most severe consequences of malaria are concentrated in the youngest age groups, and that the burden of malaria shifts towards younger age groups with increasing transmission intensity. Recently observed declines in malaria transmission intensity are likely to result in the burden of malaria shifting towards older children. Nevertheless, while the aim of malaria control remains the prevention of the most severe forms of malaria disease and death [56], targeting malaria interventions to very young children will produce important benefits. Integrated malaria strategies need to be tailored to the epidemiological context, and to be able to respond to changes in malaria transmission over time. As malaria control improves, the median age of severe disease manifestations will increase. Nevertheless, a disproportionate number of malaria deaths will continue to occur in the very young and it is still worth targeting specific control strategies at this age group.

## Supporting Information

Table S1 Sources used to allocate clinical malaria studies to a matrix of intensity and seasonality of malaria.
(0.45 MB DOC)

Table S2 Sources used to allocate studies on hospital admissions with malaria to a matrix of intensity and seasonality of malaria.
(0.51 MB DOC)

Table S3 Sources used to allocate studies of malaria-diagnosed deaths to a matrix of intensity and seasonality of malaria.
(0.27 MB DOC)
