# Supplementary material for: Age-Patterns of Malaria Vary with Severity, Transmission Intensity and Seasonality in Sub-Saharan Africa: A Systematic Review and Pooled Analysis
Source: PLoS One. 2010 Feb 1;5(2):e8988. doi: 10.1371/journal.pone.0008988 (PMC2813874; doi:10.1371/journal.pone.0008988)
Supplement: Table S1 — Sources used to allocate clinical malaria studies to a matrix of intensity and seasonality of malaria. (0.45 MB DOC) [file pone.0008988.s001.doc]

**Table S1 – Sources used to allocate clinical malaria studies to a matrix of intensity and seasonality of malaria**

| **Reference** | **Study site (Country)** | **Age range (months)** | **Study period** | **Seasonality category** | **EIR category (bites per person per year)** |
| --- | --- | --- | --- | --- | --- |
| Alonso *et al..*[1] and Kitua *et al..*[2] | Idete (Tanzania) | 0-59.9 | Aug 1993 to Jul 1994 | Not markedly seasonal^1,3^ | >100 [3] |
| Baird *et al..*[4] | Kassena-Nankana (Ghana) | 6-23.9 | Nov 1996 to May 1997 | Markedly seasonal^2^ | >100 [5] |
| Bloland *et al.*[6] | Asembo Bay (Kenya) | 0-155.9 | Jul 1992 to Oct 1996 | Not markedly seasonal^1,2,3^ | >100 [7] |
| Bonnet *et al.*[8] | Ebolakounou (Cameroon) | 12-191.9 | Mar 1997 to Sep 1998 | Markedly seasonal^3^ | 10-100^1^ |
| Bonnet *et al.*[8] | Koundou (Cameroon) | 0-191.9 | Mar 2005 to Jan 2007 | Not markedly seasonal^3^ | >100^1^ |
| Chandramohan *et al.*[9] | Kassena-Nankana (Ghana) | 2-30.9 | Sep 2000 to Jun 2004 | Markedly seasonal^1,2^ | >100 [5] |
| Cisse *et al.*[10] | Niakhar (Senegal) | 10-83.9 | Sep to Dec 2003 | Markedly seasonal^†3^ | 10-100 [11] |
| Davis *et al.*[12] | Kampala (Uganda) | 18-131.9 | Nov 2004 to Sep 2006 | Not markedly seasonal^3^ | <10^1^ |
| Diallo *et al.*[13] | South District of Dakar (Senegal) | 0-167.9 | Jun 1994 to May 1995 | Markedly seasonal^†3^ | <10 [14] |
| Diallo *et al.*[14] | Central District of Dakar (Senegal) | 0-167.9 | Mar 1996 to Feb 1997 | Markedly seasonal^†3^ | <10^1^ |
| Dicko *et al.*[15] | Sotuba (Mali) | 0-179.9 | Jul to Dec 1999 | Markedly seasonal^1^ | 10-100 [15] |
| Dicko *et al.*[15] | Sotuba (Mali) | 0-179.9 | Jul to Dec 2000 | Markedly seasonal^1^ | <10 [15] |
| Dicko *et al.*[15] | Donéguébougou (Mali) | 0-179.9 | Jul to Dec 1999 & Jul to Dec 2000 | Markedly seasonal^1^ | >100 [15] |
| Greenwood *et al.*[16] | Farafenni (The Gambia) | 0-83.9 | Apr 1982 to Mar 1983 | Markedly seasonal^2^ | 10-100 [17] |
| Grobusch *et al.*[18] | Lambarene (Gabon) | 0-27.9 | Dec 2002 to Aug 2006 | Not markedly seasonal^2,3^ [19] | 10-100^1^[19] |
| Guinovart *et al.*[20] | Manhica (Mozambique) | 0-179.9 | Jun 2003 to May 2005 | Not markedly seasonal^2^ | 10-100 [21] |
| Henning *et al.*[22] | Ifakara (Tanzania) | 0-71.9 | March 1998 to Jan 1999 | Not markedly seasonal^2^ | 10-100 [23] |
| Henry *et al.*[24] | Korhogoro (Cote d'Ivoire) | 0-191.9 | Mar 1997 to Jan 1998 | Not markedly seasonal^3^ | >100 [25] |
| Kobbe *et al..* [26] | Afigya Sekyere (Ghana) | 3-24.9 | Jan 2003 to Sep 2005 | Not markedly seasonal^1^ | >100^1^ |
| Lusingu *et al.*[27] | Tanga Region (Tanzania) | 0-239.9 | Apr to Sep 2001 | Not markedly seasonal^1,3^ [28] | >100 [29-31] |
| Maxwell *et al.*[32] | Lowland Muheza (Tanzania) | 6-143.9 | Jul 1998 to Aug 1999 | Not markedly seasonal^3^ [27,28] | >100 [29-31] |
| Maxwell *et al.*[32] | Highland Muheza (Tanzania) | 6-143.9 | Jul 1998 to Aug 1999 | Not markedly seasonal^3^ [27,28] | 10-100 [33] |
| McGuinness *et al.*[34] and Wagner *et al.*[35] | Prampram (Ghana) | 0-35.9 | May 1994 to Mar 1998 | Not markedly seasonal [35] | <10 [35] |
| Rogier *et al.*[36] | Dielmo (Senegal) | 0-179.9 | May 1990 to Nov 1993 | Not markedly seasonal^1^ [37,38] | >100 [38] |
| Saute *et al.*[39] | Manhica (Mozambique) | 2-107.9 | Dec 1996 to Jul 1999 | Not markedly seasonal^2^ | 10-100 [21] |
| Schellenberg *et al.*[40] | Ifakara (Tanzania) | 0-59.9 | Jul 2000 to Jun 2001 | Not markedly seasonal^3^ | 10-100 [23] |
| Thompson *et al.*[41] | Maputo (Mozambique) | 0-47.9 | Dec 1992 to Jun 1995 | Markedly seasonal^1,2,3^ | 10-100 [42] |
| Velema *et al.*[43] | Atlantic Coast (Benin) | 0-35.9 | Apr to Dec 1989 | Not markedly seasonal^3^ | >100^§^[44] |
| Ye *et al.*[45] | Nouna District (Burkina Faso) | 6-59.9 | Dec 2003 to Nov 2004 | Markedly seasonal^3^ [46] | >100^†^ [46] |

Unpublished sources of data: ^1^ Authors’ description in paper or personal communication with authors; ^2^ Seasonality analysis [47]; ^3^ MARA seasonality maps [48]; ^§^ Log-linear relationship between parasite prevalence and EIR with source of prevalence data cited subsequently; ^†^ Local/Expert opinion

**Bibliography**

1. Alonso PL, Smith T, Armstrong Schellenberg JRM, Masanja H, Mwankusye S, et al. (1994) Randomised trial of efficacy of SPf66 vaccine against Plasmodium falciparum malaria in children in southern Tanzania. Lancet (British edition) 344: 1175-1181.

2. Kitua AY, Smith T, Alonso PL, Masanja H, Urassa H, et al. (1996) Plasmodium falciparum malaria in the first year of life in an area of intense and perennial transmission. Tropical Medicine and International Health 1: 475-484.

3. Charlwood JD, Smith T, Lyimo E, Kitua AY, Masanja H, et al. (1998) Incidence of Plasmodium falciparum infection in infants in relation to exposure to sporozoite-infected anophelines. Am J Trop Med Hyg 59: 243-251.

4. Baird JK, Owusu AS, Utz GC, Koram K, Barcus MJ, et al. (2002) Seasonal malaria attack rates in infants and young children in northern Ghana. Am J Trop Med Hyg 66: 280-286.

5. Appawu M, Owusu-Agyei S, Dadzie S, Asoala V, Anto F, et al. (2004) Malaria transmission dynamics at a site in northern Ghana proposed for testing malaria vaccines. Trop Med Int Health 9: 164-170.

6. Bloland PB, Boriga DA, Ruebush TK, McCormick JB, Roberts JM, et al. (1999) Longitudinal cohort study of the epidemiology of malaria infections in an area of intense malaria transmission II. Descriptive epidemiology of malaria infection and disease among children. Am J Trop Med Hyg 60: 641-648.

7. Beier JC, Perkins PV, Onyango FK, Gargan TP, Oster CN, et al. (1990) Characterization of malaria transmission by Anopheles (Diptera: Culicidae) in western Kenya in preparation for malaria vaccine trials. J Med Entomol 27: 570-577.

8. Bonnet S, Paul RE, Gouagna C, Safeukui I, Meunier JY, et al. (2002) Level and dynamics of malaria transmission and morbidity in an equatorial area of South Cameroon. Trop Med Int Health 7: 249-256.

9. Chandramohan D, Owusu-Agyei S, Carneiro I, Awine T, Amponsa-Achiano K, et al. (2005) Cluster randomised trial of intermittent preventive treatment for malaria in infants in area of high, seasonal transmission in Ghana. Bmj 331: 727-733.

10. Cisse B, Sokhna C, Boulanger D, Milet J, Ba el H, et al. (2006) Seasonal intermittent preventive treatment with artesunate and sulfadoxine-pyrimethamine for prevention of malaria in Senegalese children: a randomised, placebo-controlled, double-blind trial. Lancet 367: 659-667.

11. Robert V, Dieng H, Lochouran L, Traore SF, Trape JF, et al. (1998) [Malaria transmission in the rural zone of Niakhar, Senegal]. Trop Med Int Health 3: 667-677.

12. Davis JC, Clark TD, Kemble SK, Talemwa N, Njama-Meya D, et al. (2006) Longitudinal study of urban malaria in a cohort of Ugandan children: description of study site, census and recruitment. Malar J 5: 18.

13. Diallo S, Ndir O, Faye O, Diop BM, Dieng Y, et al. (1998) [Malaria in the southern sanitary district of Dakar (Senegal). 1. Parasitemia and malarial attacks]. Bull Soc Pathol Exot 91: 208-213.

14. Diallo S, Konate L, Ndir O, Dieng T, Dieng Y, et al. (2000) [Malaria in the central health district of Dakar (Senegal). Entomological, parasitological and clinical data]. Sante 10: 221-229.

15. Dicko A, Sagara I, Diemert D, Sogoba M, Niambele MB, et al. (2007) Year-to-year variation in the age-specific incidence of clinical malaria in two potential vaccine testing sites in Mali with different levels of malaria transmission intensity. Am J Trop Med Hyg 77: 1028-1033.

16. Greenwood BM, Bradley AK, Greenwood AM (1987) Mortality and morbidity from malaria among children in a rural area of the Gambia, West Africa. Transactions of the Royal Society of Tropical Medicine and Hygiene 81: 478-486.

17. Lindsay SW, Shenton FC, Snow RW, Greenwood BM (1989) Responses of Anopheles gambiae complex mosquitoes to the use of untreated bednets in The Gambia. Med Vet Entomol 3: 253-262.

18. Grobusch MP, Lell B, Schwarz NG, Gabor J, Dornemann J, et al. (2007) Intermittent preventive treatment against malaria in infants in Gabon--a randomized, double-blind, placebo-controlled trial. J Infect Dis 196: 1595-1602.

19. Klein Klouwenberg PM, Oyakhirome S, Schwarz NG, Glaser B, Issifou S, et al. (2005) Malaria and asymptomatic parasitaemia in Gabonese infants under the age of 3 months. Acta Trop 95: 81-85.

20. Guinovart C, Bassat Q, Sigauque B, Aide P, Sacarlal J, et al. (2008) Malaria in rural Mozambique. Part I: children attending the outpatient clinic. Malar J 7: 36.

21. Aranda C, Aponte JJ, Saute F, Casimiro S, Pinto J, et al. (2005) Entomological characteristics of malaria transmission in Manhica, a rural area in southern Mozambique. J Med Entomol 42: 180-186.

22. Henning L, Schellenberg D, Smith T, Henning D, Alonso P, et al. (2004) A prospective study of Plasmodium falciparum multiplicity of infection and morbidity in Tanzanian children. Trans R Soc Trop Med Hyg 98: 687-694.

23. Drakeley C, Schellenberg D, Kihonda J, Sousa CA, Arez AP, et al. (2003) An estimation of the entomological inoculation rate for Ifakara: a semi-urban area in a region of intense malaria transmission in Tanzania. Trop Med Int Health 8: 767-774.

24. Henry MC, Rogier C, Nzeyimana I, Assi SB, Dossou-Yovo J, et al. (2003) Inland valley rice production systems and malaria infection and disease in the savannah of Cote d'Ivoire. Trop Med Int Health 8: 449-458.

25. Dossou-Yovo JD, Diarrassouba S, Henry MC, Briet OJT, Akodo E, et al. (Unpublished) Rice production systems and malaria in the savanna of Cote d'Ivoire: entomological input. Institut Pierre Richet, Bouake, Cote d'Ivoire.

26. Kobbe R, Kreuzberg C, Adjei S, Thompson B, Langefeld I, et al. (2007) A randomized controlled trial of extended intermittent preventive antimalarial treatment in infants. Clin Infect Dis 45: 16-25.

27. Lusingu JP, Vestergaard LS, Mmbando BP, Drakeley CJ, Jones C, et al. (2004) Malaria morbidity and immunity among residents of villages with different Plasmodium falciparum transmission intensity in North-Eastern Tanzania. Malar J 3: 26.

28. Massaga JJ, Kitua AY, Lemnge MM, Akida JA, Malle LN, et al. (2003) Effect of intermittent treatment with amodiaquine on anaemia and malarial fevers in infants in Tanzania: a randomised placebo-controlled trial. Lancet 361: 1853-1860.

29. Curtis CF, Maxwell CA, Finch RJ, Njunwa KJ (1998) A comparison of use of a pyrethroid either for house spraying or for bednet treatment against malaria vectors. Trop Med Int Health 3: 619-631.

30. Magesa SM, Wilkes TJ, Mnzava AE, Njunwa KJ, Myamba J, et al. (1991) Trial of pyrethroid impregnated bednets in an area of Tanzania holoendemic for malaria. Part 2. Effects on the malaria vector population. Acta Trop 49: 97-108.

31. Maxwell CA, Myamba J, Njunwa KJ, Greenwood BM, Curtis CF (1999) Comparison of bednets impregnated with different pyrethroids for their impact on mosquitoes and on re-infection with malaria after clearance of pre-existing infections with chlorproguanil-dapsone. Trans R Soc Trop Med Hyg 93: 4-11.

32. Maxwell CA, Chambo W, Mwaimu M, Magogo F, Carneiro IA, et al. (2003) Variation of malaria transmission and morbidity with altitude in Tanzania and with introduction of alphacypermethrin treated nets. Malar J 2: 28.

33. Ellman R, Maxwell C, Finch R, Shayo D (1998) Malaria and anaemia at different altitudes in the Muheza district of Tanzania: childhood morbidity in relation to level of exposure to infection. Ann Trop Med Parasitol 92: 741-753.

34. McGuinness D, Koram K, Bennett S, Wagner G, Nkrumah F, et al. (1998) Clinical case definitions for malaria: clinical malaria associated with very low parasite densities in African infants. Trans R Soc Trop Med Hyg 92: 527-531.

35. Wagner G, Koram K, McGuinness D, Bennett S, Nkrumah F, et al. (1998) High incidence of asymptomatic malaria infections in a birth cohort of children less than one year of age in Ghana, detected by multicopy gene polymerase chain reaction. American Journal of Tropical Medicine and Hygiene 59: 115-123.

36. Rogier C, Trape JF (1993) Malaria attacks in children exposed to high transmission: who is protected? Trans R Soc Trop Med Hyg 87: 245-246.

37. Rogier C, Ly AB, Tall A, Cisse B, Trape JF (1999) Plasmodium falciparum clinical malaria in Dielmo, a holoendemic area in Senegal: no influence of acquired immunity on initial symptomatology and severity of malaria attacks. Am J Trop Med Hyg 60: 410-420.

38. Trape JF, Rogier C, Konate L, Diagne N, Bouganali H, et al. (1994) The Dielmo project: a longitudinal study of natural malaria infection and the mechanisms of protective immunity in a community living in a holoendemic area of Senegal. Am J Trop Med Hyg 51: 123-137.

39. Saute F, Aponte J, Ahmeda J, Ascaso C, Vaz N, et al. (2003) Malaria in southern Mozambique: incidence of clinical malaria in children living in a rural community in Manhica district. Trans R Soc Trop Med Hyg 97: 655-660.

40. Schellenberg D, Aponte J, Kahigwa E, Mshinda H, Tanner M, et al. (2003) The incidence in children of clinical malaria detected by active case detection in Ifakara, southern Tanzania. Trans R Soc Trop Med Hyg 97: TR/2002/020410.

41. Thompson R, Begtrup K, Cuamba N, Dgedge M, Mendis C, et al. (1997) The Matola malaria project: a temporal and spatial study of malaria transmission and disease in a suburban area of Maputo, Mozambique. Am J Trop Med Hyg 57: 550-559.

42. Mendis C, Jacobsen JL, Gamage-Mendis A, Bule E, Dgedge M, et al. (2000) Anopheles arabiensis and An. funestus are equally important vectors of malaria in Matola coastal suburb of Maputo, southern Mozambique. Med Vet Entomol 14: 171-180.

43. Velema JP, Alihonou EM, Chippaux JP, Van Boxel Y, Gbedji E, et al. (1991) Malaria morbidity and mortality in children under three years of age on the coast of Benin, West Africa. Transactions of the Royal Society of Tropical Medicine and Hygiene 85: 430-435.

44. Akogbeto M, Modiano D, Bosman A (1992) Malaria transmission in the lagoon area of Cotonou, Benin. Parassitologia 34: 147-154.

45. Ye Y, Kyobutungi C, Louis VR, Sauerborn R (2007) Micro-epidemiology of Plasmodium falciparum malaria: Is there any difference in transmission risk between neighbouring villages? Malar J 6: 46.

46. Becher H, Kynast-Wolf G, Sie A, Ndugwa R, Ramroth H, et al. (2008) Patterns of malaria: cause-specific and all-cause mortality in a malaria-endemic area of west Africa. Am J Trop Med Hyg 78: 106-113.

47. Roca-Feltrer A, Armstrong Schellenberg JR, Smith L, Carneiro I (2009) A simple method for defining malaria seasonality. Malar J 8: 276.

48. Mapping Malaria Risk in Africa (2008) Duration of Malaria Transmission Season.
