# Supplementary material for: Age-Patterns of Malaria Vary with Severity, Transmission Intensity and Seasonality in Sub-Saharan Africa: A Systematic Review and Pooled Analysis
Source: PLoS One. 2010 Feb 1;5(2):e8988. doi: 10.1371/journal.pone.0008988 (PMC2813874; doi:10.1371/journal.pone.0008988)
Supplement: Table S2 — Sources used to allocate studies on hospital admissions with malaria to a matrix of intensity and seasonality of malaria. (0.51 MB DOC) [file pone.0008988.s002.doc]

**Table S2 – Sources used to allocate studies on hospital admissions with malaria to a matrix of intensity and seasonality of malaria**

| **Reference** | **Study site (Country)** | **Age range (months)** | **Study period** | **Seasonality category** | **EIR category (bites per person per year)** |
| --- | --- | --- | --- | --- | --- |
| Bassat *et al.* [1] | Manhica District Hospital (Mozambique) | 0-179.9 | Jun 2003 to May 2005 | Not markedly seasonal^2^ | 10-100 [2] |
| Boulard *et al.*[3] | Cotonou Hospital (Benin) | 0-179.9 | Apr 1988 to Mar 1989 | Not markedly seasonal^3^ | 10-100 [4] |
| Camara *et al.*[5] | Albert Royer Children’s Hospital, Dakar (Senegal) | 0-191.9 | Jul 1998 to Dec 1999 | Markedly seasonal^3†^ [6,7] | <10 [8] |
| Giha *et al.* [9] | Geradif (Sudan) | 10-179.9 | Oct 2000 to Dec 2001 | Markedly seasonal^1^ [10] | <10 [11] |
| Guiguemde  *et al.* [12] | Ouagadougou University Hospital (Burkina Faso) | 0-179.9 | Jan to Dec 1988 | Markedly seasonal^1^ [13] | 10-100 [14] |
| Imbert *et al.* [7] | Hopital Principal Dakar (Senegal) | 0-191.9 | Jan 1990 to Feb 1996 | Markedly seasonal^1,3†^ | <10 [8] |
| Imbert *et al.* [6] | Hopital Principal Dakar (Senegal) | 0-191.9 | Oct 1997 to Mar 1999 | Markedly seasonal^1,3†^ | <10 [8] |
| Jallow *et al.* [15] | Royal Victoria Teaching Hospital, Banjul (The Gambia) | 0-179.9 | Dec 1996 to Nov 2000 | Markedly seasonal^2,3^ [16,17] | <10 [18] |
| Kazembe *et al.* [19] | Zomba District Hospital (Malawi) | 1-179.9 | Jan 2002 to Dec 2003 | Not markedly seasonal^†^ | 10-100^§^ [20] |
| Koko *et al.* [21] | Libreville (Gabon) | 0-191.9 | Jan to Dec 1992 | Not markedly seasonal^1,2,3^ | 10-100^§^ [22] |
| Maitland *et al.* [23] | Kilifi District Hospital (Kenya) | 0-167.9 | Sep 1999 to Dec 2000 | Not markedly seasonal^2^ [24] | 10-100 [25,26] |
| Mockenhaupt *et al.*[27] | Tamale (Ghana) | 6-107.9 | Aug to Nov 2002 | Not markedly seasonal^1,3^ | >100^1^ |
| Modiano *et al.*[28] | Ouagadougou University Hospital (Burkina Faso) | 0-179.9 | Jun to Oct 1996 | Markedly seasonal^1,3^ [13] | 10-100 [14] |
| Modiano *et al.*[28] | Sorou & Nayala District Hospitals (Burkina Faso) | 0-167.9 | Jun to Oct 1993 and 1994 | Markedly seasonal^1,3^ [13] | >100 [14,29] |
| Mulumba *et al.*[30] | PHK, Kinshasa (DRC) | 1-143.9 | Sep to Dec 2004 | Not markedly seasonal^3^ | 10-100 [31] |
| Mulumba *et al.*[30] | HJH, Kinshasa (DRC) | 2-155.9 | Sep to Dec 2004 | Not markedly seasonal^3^ | 10-100 [31] |
| Olanrewaju *et al.*[32] | Ilorin (Nigeria) | 0-191.9 | Jan to Dec 1998 | Not markedly seasonal^1,†^ | 10-100^†^ |
| Reyburn *et al.* [33] | Huruma (Tanzania) | 3-155.9 | Feb 2002 to Feb 2003 | Markedly seasonal^2^ | <10^1^ |
| Reyburn *et al.* [33] | KCMC & Mawenzi Hospitals, Moshi (Tanzania) | 0-167.9 | Feb 2002 to Feb 2003 | Not markedly seasonal^2^ | 10-100^1^ |
| Reyburn *et al.* [33] | Kibosho (Tanzania) | 0-167.9 | Feb 2002 to Feb 2003 | Markedly seasonal^2,3^ | <10^1^ |
| Reyburn *et al.* [33] | Same (Tanzania) | 0-167.9 | Feb 2002 to Feb 2003 | Markedly seasonal^2,3^ | 10-100^1^ |
| Reyburn *et al.* [33] | Teule (Tanzania) | 0-167.9 | Feb 2002 to Feb 2003 | Not markedly seasonal^2,3^ | >100^1^ |
| Rodrigues *et al.* [34] | Simao Mendes National Hospital, Bissau (Guinea Bissau) | 1-59.9 | May 2003 to May 2004 | Markedly seasonal^1^ | <10^§1^ |
| Schapira *et al.*[35] | Maputo Central Hospital (Mozambique) | 7-95.9 | Feb to Jun 1990 | Markedly seasonal^2,3^[36] | 10-100 [37] |
| Schellenberg *et al.*[38] | Ifakara (Tanzania) | 0-179.9 | Jan to Dec 1995 and 2000 | Not markedly seasonal^2,3 †^ | 10-100 [39] |
| Slutsker *et al.*[40] | Mangochi District Hospital (Malawi) | 0-191.9 | Mar 1990 to Feb 1992 | Not markedly seasonal^1,2^ [41] | 10-100^§^ [42] |
| Slutsker *et al.*[40] | Queen Elizabeth Hospital, Blantyre (Malawi) | 0-191.9 | Mar 1990 to Feb 1991 | Not markedly seasonal^1,2^[41] | 10-100^§^ [43] |
| Snow *et al.*[44] | Kilifi (Kenya) | 0-155.9 | May 1989 to Apr 1992 | Not markedly seasonal^2^ [24] | 10-100 [25,26] |
| Taylor *et al.*[45] | Lambarene (Gabon) | 0-167.9 | Dec 2000 to Apr 2005 | Not markedly seasonal^2,3^ [46] | 10-100[46] |
| Taylor *et al.*[45] | Libreville (Gabon) | 2-155.9 | Dec 2000 to Apr 2005 | Not markedly seasonal^2,3^ [21] | 10-100^§^ [22] |
| Taylor *et al.*[45] | Royal Victoria Teaching Hospital, Banjul (The Gambia) | 0-167.9 | Dec 2000 to Apr 2005 | Markedly seasonal^2,3^ [16,17] | <10 [18] |
| Taylor *et al.*[45] | Komfo Anokye Teaching Hospital, Kumasi (Ghana) | 0-167.9 | Dec 2000 to Apr 2005 | Not markedly seasonal^2,3^ | 10-100^†^ |
| Taylor *et al.*[45] | Kilifi District Hospital (Kenya) | 0-167.9 | Dec 2000 to Apr 2005 | Not markedly seasonal^2^ [24] | 10-100 [25,26] |
| Taylor *et al.*[45] | Queen Elizabeth Teaching Hospital, Blantyre (Malawi) | 0-167.9 | Dec 2000 to Apr 2005 | Not markedly seasonal^2†^ [40,41] | 10-100^§^ [43] |
| Thuma *et al.*[47] | Macha Mission Hospital (Zambia) | 1-83.9 | Jan 2003 to Dec 2004 | Markedly seasonal^1,2,3^ [48] | 10-100^1^ [49] |
| Varandas *et al.* [50] | Maputo Central Hospital (Mozambique) | 6-71.9 | Feb 1995 to Apr 1997 | Markedly seasonal^2,3^[36] | 10-100 [37] |

Unpublished sources of data: ^1^ Authors’ description in paper or personal communication with authors; ^2^ Sites categorised according to seasonality analysis [51]; ^3^ MARA seasonality maps [52]; ^§^ Log-linear relationship between parasite prevalence and EIR with source of prevalence data cited subsequently; ^†^ Local/Expert opinion

**Bibliography**

1. Bassat Q, Guinovart C, Sigauque B, Aide P, Sacarlal J, et al. (2008) Malaria in rural Mozambique. Part II: children admitted to hospital. Malar J 7: 37.

2. Aranda C, Aponte JJ, Saute F, Casimiro S, Pinto J, et al. (2005) Entomological characteristics of malaria transmission in Manhica, a rural area in southern Mozambique. J Med Entomol 42: 180-186.

3. Boulard JC, Chippaux JP, Ayivi B, Akogbeto M, Massougbodji A, et al. (1990) Study of malarial morbidity in a hospital paediatric service in Benin (West Africa) in 1988 and 1989. Medecine Tropicale 50: 315-320.

4. Akogbeto M (2000) [Lagoonal and coastal malaria at Cotonou: entomological findings]. Sante 10: 267-275.

5. Camara B, Diouf S, Diagne I, Fall L, Ba A, et al. (2003) Severe malaria in children in a Senegal hospital setting. Médecine et Maladies Infectieuses 33: 45-48.

6. Imbert P, Gerardin P, Rogier C, Ka AS, Jouvencel P, et al. (2002) Severe falciparum malaria in children: a comparative study of 1990 and 2000 WHO criteria for clinical presentation, prognosis and intensive care in Dakar, Senegal. Trans R Soc Trop Med Hyg 96: 278-281.

7. Imbert P, Sartelet I, Rogier C, Ka S, Baujat G, et al. (1997) Severe malaria among children in a low seasonal transmission area, Dakar, Senegal: influence of age on clinical presentation. Trans R Soc Trop Med Hyg 91: 22-24.

8. Diallo S, Konate L, Ndir O, Dieng T, Dieng Y, et al. (2000) [Malaria in the central health district of Dakar (Senegal). Entomological, parasitological and clinical data]. Sante 10: 221-229.

9. Giha HA, El-Ghazali G, A-Elgadir TME, A-Elbasit IE, Eltahir EM, et al. (2005) Clinical pattern of severe Plasmodium falciparum malaria in Sudan in an area characterized by seasonal and unstable malaria transmission. Transactions of the Royal Society of Tropical Medicine and Hygiene 99: 243-251.

10. Giha HA, Rosthoj S, Dodoo D, Hviid L, Satti GM, et al. (2000) The epidemiology of febrile malaria episodes in an area of unstable and seasonal transmission. Trans R Soc Trop Med Hyg 94: 645-651.

11. Hamad AA, Nugud Ael H, Arnot DE, Giha HA, Abdel-Muhsin AM, et al. (2002) A marked seasonality of malaria transmission in two rural sites in eastern Sudan. Acta Trop 83: 71-82.

12. Guiguemde TR, Dabire E, Coulibaly CO, Pare J, Kam LK (1991) Etude de la mortalite palustre dans le service de pediatrie de l'hopital de Ouagadougou (Burkina Faso). Bull Soc Pathol Exot 84: 338-339.

13. Bolad A, Nebié I, Esposito F, Berzins K (2004) The use of impregnated curtains does not affect antibody responses against Plasmodium falciparum and complexity of infecting parasite populations in children from Burkina Faso. Acta Tropica 90: 237-247.

14. Rossi P, Belli A, Mancini L, Sabatinelli G (1986) [A longitudinal entomologic survey on the transmission of malaria in Ouagadougou (Burkina Faso)]. Parassitologia 28: 1-15.

15. Jallow M MalariaGEN project.

16. Greenwood BM, Greenwood AM, Bradley AK (1988) Comparison of two strategies for control of malaria within a primary health care programme in the Gambia. Lancet i: 1121-1127.

17. Snow RW, Rowan KM, Greenwood BM (1987) A trial of permethrin-treated bed nets in the prevention of malaria in Gambian children. Trans R Soc Trop Med Hyg 81: 563-567.

18. Lindsay SW, Shenton FC, Snow RW, Greenwood BM (1989) Responses of Anopheles gambiae complex mosquitoes to the use of untreated bednets in The Gambia. Med Vet Entomol 3: 253-262.

19. Kazembe LN, Kleinschmidt I, Sharp BL (2006) Patterns of malaria-related hospital admissions and mortality among Malawian children: an example of spatial modelling of hospital register data. Malar J 5: 93.

20. Kazembe LN, Kleinschmidt I, Holtz TH, Sharp BL (2006) Spatial analysis and mapping of malaria risk in Malawi using

point-referenced prevalence of infection data. International Journal of Health Geographics 5: 9.

21. Koko J, Dufillot D, Zima-Ebeyard AM, Duong TH, Gahouma D, et al. (1999) Clinical and epidemiological aspects of malaria in children in Libreville, Gabon. Medecine d'Afrique Noire 46: 10-14.

22. Merlin M, Dupont A, Josse R, Delaporte E, Cheringou H, et al. (1990) [Epidemiologic aspects of malaria in Gabon]. Med Trop (Mars) 50: 39-46.

23. Maitland K, Levin M, English M, Mithwani S, Peshu N, et al. (2003) Severe P. falciparum malaria in Kenyan children: evidence for hypovolaemia. Qjm 96: 427-434.

24. Snow RW, Bastos de Azevedo I, Lowe BS, Kabiru EW, Nevill CG, et al. (1994) Severe childhood malaria in two areas of markedly different falciparum transmission in east Africa. Acta-Trop 57: 289-300.

25. Mbogo CM, Mwangangi JM, Nzovu J, Gu W, Yan G, et al. (2003) Spatial and temporal heterogeneity of Anopheles mosquitoes and Plasmodium falciparum transmission along the Kenyan coast. Am J Trop Med Hyg 68: 734-742.

26. Mbogo CN, Snow RW, Khamala CP, Kabiru EW, Ouma JH, et al. (1995) Relationships between Plasmodium falciparum transmission by vector populations and the incidence of severe disease at nine sites on the Kenyan coast. Am J Trop Med Hyg 52: 201-206.

27. Mockenhaupt FP, Ehrhardt S, Burkhardt J, Bosomtwe SY, Laryea S, et al. (2004) Manifestation and outcome of severe malaria in children in northern Ghana. Am J Trop Med Hyg 71: 167-172.

28. Modiano D, Sirima BS, Sawadogo A, Sanou I, Pare J, et al. (1998) Severe malaria in Burkina Faso: influence of age and transmission level on clinical presentation. American Journal of Tropical Medicine and Hygiene 59: 539-542.

29. Pietra Y, Procacci PG, Sabatinelli G, Kumlien S, Lamizana L, et al. (1991) [Impact of utilization of permethrin impregnated curtains on malaria in a rural zone of high transmission in Burkina Faso]. Bull Soc Pathol Exot 84: 375-385.

30. Mulumba MP, Ilunga I, Bankoto A, Kamba VD (Submitted) Radioscopie du polymorphisme clinique de l'access de pernicieux palustre de l'enfant a Kinshasa. Congo Medical Journal.

31. Coene J (1993) Malaria in urban and rural Kinshasa: the entomological input. Med Vet Entomol 7: 127-137.

32. Olanrewaju WI, Johnson AWBR (2001) Malaria in children in Ilorin, Nigeria. East African Medical Journal 78: 131-134.

33. Reyburn H, Mbatia R, Drakeley C, Bruce J, Carneiro I, et al. (2005) Association of transmission intensity and age with clinical manifestations and case fatality of severe Plasmodium falciparum malaria. JAMA 293: 1461-1470.

34. Rodrigues A, Schellenberg JA, Kofoed PE, Aaby P, Greenwood B (2008) Changing pattern of malaria in Bissau, Guinea Bissau. Trop Med Int Health 13: 410-417.

35. Schapira A, Solomon T, Julien M, Macome A, Parmar N, et al. (1993) Comparison of intramuscular and intravenous quinine for the treatment of severe and complicated malaria in children. Trans R Soc Trop Med Hyg 87: 299-302.

36. Thompson R, Begtrup K, Cuamba N, Dgedge M, Mendis C, et al. (1997) The Matola malaria project: a temporal and spatial study of malaria transmission and disease in a suburban area of Maputo, Mozambique. Am J Trop Med Hyg 57: 550-559.

37. Mendis C, Jacobsen JL, Gamage-Mendis A, Bule E, Dgedge M, et al. (2000) Anopheles arabiensis and An. funestus are equally important vectors of malaria in Matola coastal suburb of Maputo, southern Mozambique. Med Vet Entomol 14: 171-180.

38. Schellenberg D, Menendez C, Aponte J, Guinovart C, Mshinda H, et al. (2004) The Changing Epidemiology of Malaria in Ifakara Town, southern Tanzania. Tropical Medicine and International Health 9: 68-76.

39. Drakeley C, Schellenberg D, Kihonda J, Sousa CA, Arez AP, et al. (2003) An estimation of the entomological inoculation rate for Ifakara: a semi-urban area in a region of intense malaria transmission in Tanzania. Trop Med Int Health 8: 767-774.

40. Slutsker L, Taylor TE, Wirima JJ, Steketee RW (1994) In-hospital morbidity and mortality due to malaria-associated severe anaemia in two areas of Malawi with different patterns of malaria infection. Trans R Soc Trop Med Hyg 88: 548-551.

41. Reed SC, Wirima JJ, Steketee RW (1994) Risk factors for anemia in young children in rural Malawi. Am J Trop Med Hyg 51: 170-174.

42. Yeudall F, Gibson RS, Kayira C, Umar E (2002) Efficacy of a multi-micronutrient dietary intervention based on haemoglobin, hair zinc concentrations, and selected functional outcomes in rural Malawian children. Eur J Clin Nutr 56: 1176-1185.

43. Holtz TH, Marum LH, Mkandala C, Chizani N, Roberts JM, et al. (2002) Insecticide-treated bednet use, anaemia, and malaria parasitaemia in Blantyre District, Malawi. Tropical Medicine and International Health 7: 220-230.

44. Snow RW, Armstrong Schellenberg JRM, Peshu N, Forster D, Newton CRJC, et al. (1993) Periodicity and space-time clustering of severe childhood malaria on the coast of Kenya. Transactions of the Royal Society of Tropical Medicine and Hygiene 87: 386-390.

45. Taylor T, Olola C, Valim C, Agbenyega T, Kremsner P, et al. (2006) Standardized data collection for multi-center clinical studies of severe malaria in African children: establishing the SMAC network. Trans R Soc Trop Med Hyg 100: 615-622.

46. Klein Klouwenberg PM, Oyakhirome S, Schwarz NG, Glaser B, Issifou S, et al. (2005) Malaria and asymptomatic parasitaemia in Gabonese infants under the age of 3 months. Acta Trop 95: 81-85.

47. Thuma PE Malaria admissions to Macha Mission Hospital Zambia (2003-2004).

48. Biemba G, Dolmans D, Thuma PE, Weiss G, Gordeuk VR (2000) Severe anaemia in Zambian children with Plasmodium falciparum malaria. Tropical Medicine and International Health 5: 9-16.

49. Siachinji V, Thuma P, Mulenga M (2004) Entomologic studies in Southern Province of Zambia to determine major vector of

malaria. THE FOURTH AMANET BIENNIAL CONFERENCE. Arusha, Tanzania.

50. Varandas L, Julien M, Gomes A, Rodrigues P, Van Lerberghe W, et al. (2001) A randomised, double-blind, placebo-controlled clinical trial of vitamin A in severe malaria in hospitalised Mozambican children. Ann Trop Paediatr 21: 211-222.

51. Roca-Feltrer A, Armstrong Schellenberg JR, Smith L, Carneiro I (2009) A simple method for defining malaria seasonality. Malar J 8: 276.

52. Mapping Malaria Risk in Africa (2008) Duration of Malaria Transmission Season.
