# Supplementary material for: Age-Patterns of Malaria Vary with Severity, Transmission Intensity and Seasonality in Sub-Saharan Africa: A Systematic Review and Pooled Analysis
Source: PLoS One. 2010 Feb 1;5(2):e8988. doi: 10.1371/journal.pone.0008988 (PMC2813874; doi:10.1371/journal.pone.0008988)
Supplement: Table S3 — Sources used to allocate studies of malaria-diagnosed deaths to a matrix of intensity and seasonality of malaria. (0.27 MB DOC) [file pone.0008988.s003.doc]

**Table S3 – Sources used to allocate studies of malaria-diagnosed deaths to a matrix of intensity and seasonality of malaria**

| **Reference** | **Study site (Country)** | **Age range (months)** | **Study period** | **Seasonality category** | **EIR category (bites per person per year)** |
| --- | --- | --- | --- | --- | --- |
| Abdullah *et al.*[1] | Kisumu (Kenya) | 0-179.9 | Aug 2001 to Sep 2005 | Not markedly seasonal^2,3^ [2] | >100 [3] |
| Abdullah *et al.*[1] | Navrongo (Ghana) | 0-179.9 | Nov 2001 to Oct 2004 | Markedly seasonal^2^ | >100 [4] |
| Abdullah *et al.*[1] | Oubritenga (Burkina Faso) | 0-179.9 | Aug 2001 to Sep 2005 | Markedly seasonal [5] | >100 [5] |
| Abdullah *et al.*[1] | Rufigji (Tanzania) | 0-179.9 | Oct 2001 to Sep 2004 | Not markedly seasonal^3^ | 10-100^†^ |
| Abdullah *et al.*[1] | Manhica (Mozambique) | 0-179.9 | Oct 2001 to Sep 2004 | Not markedly seasonal^2^ | 10-100 [6] |
| Abdullah *et al.*[1] | Kourweogo (Burkina Faso) | 0-179.9 | Jan 2002 to Dec 2003 | Markedly seasonal^1^ | >100^1^ |
| Abdullah *et al.*[1] | Ifakara (Tanzania) | 0-179.9 | Oct 2001 to Sep 2004 | Not markedly seasonal^2,3^ | 10-100 [7] |
| Alonso *et al.* [8] | South Bank (The Gambia) | 0-83.9 | Jul 1988-Jun 1990 | Markedly seasonal^2,3^ | 10-100^†^ |
| Becher [9] | Nouna (Burkina Faso) | 0-179.9 | Jan 1999 to Dec 2003 | Markedly seasonal^1,3^ | >100^1^ |
| D'Alessandro [10] | (The Gambia) | 0-119.9 | 1992-1993 | Markedly seasonal^2,3^ | 10-100^†^ |
| Etard *et al.*[11] | Niakhar (Senegal) | 0-119.9 | 1989-2000 | Markedly seasonal^1^ [12] | 10-100 [13] |
| Ghana VAST Study Team [14] | Kassena-Nankana (Ghana) | 0-59.9 | Sep 1989 to Dec 1991 | Markedly seasonal^2^ | >100 [4] |
| Greenwood *et al.*[15] | Farafenni (The Gambia) | 1-83.9 | 1982 to 1987 | Markedly seasonal^1,2,3^ | 10-100 [16] |
| Phillips-Howard *et al.*[2] | Asembo Bay (Kenya) | 0-59.9 | Apr 1997 to Mar 1998 | Not markedly seasonal^1,2,3^ | >100 [3] |
| Premji *et al.*[17] | Bagamoyo District (Tanzania) | 0-59.9 | Apr 1992 to Mar 1994 | Not markedly seasonal [18] | >100 [19,20] |
| Schumacher *et al.*[21] | Mandiana (Guinea) | 0-59.9 | Oct 1998 to Sep 1999 | Not markedly seasonal^3^ | >100^1^ |
| Snow *et al.*[22] | Kilifi (Kenya) | 0-59.9 | May 1991 to Apr 1993 | Not markedly seasonal^2^ [23] | 10-100 [24,25] |
| Trape *et al.*[12] | Bandafassi (Senegal) | 1-59.9 | Jan 1984 to Dec 1995 | Markedly seasonal^1,3^ | >100 [26] |
| Trape *et al.*[12] | Niakhar (Senegal) | 1-59.9 | Jan 1984 to Dec 1995 | Markedly seasonal^1,3^ | 10-100 [13] |

Unpublished sources of data: ^1^ Authors’ description in paper or personal communication with authors; ^2^ Seasonality analysis [27]; ^3^ MARA seasonality maps [28];

^†^ Local/Expert opinion

**Bibliography**

1. Abdullah S, Adazu K, Masanja H, Diallo D, Hodgson A, et al. (2007) Patterns of age-specific mortality in children in endemic areas of sub-Saharan Africa. Am J Trop Med Hyg 77: 99-105.

2. Phillips-Howard PA, Nahlen BL, Kolczak MS, Hightower AW, ter Kuile FO, et al. (2003) Efficacy of permethrin-treated bed nets in the prevention of mortality in young children in an area of high perennial malaria transmission in western Kenya. Am J Trop Med Hyg 68: 23-29.

3. Beier JC, Perkins PV, Onyango FK, Gargan TP, Oster CN, et al. (1990) Characterization of malaria transmission by Anopheles (Diptera: Culicidae) in western Kenya in preparation for malaria vaccine trials. J Med Entomol 27: 570-577.

4. Appawu M, Owusu-Agyei S, Dadzie S, Asoala V, Anto F, et al. (2004) Malaria transmission dynamics at a site in northern Ghana proposed for testing malaria vaccines. Trop Med Int Health 9: 164-170.

5. Cuzin-Ouattara N, Van den Broek AH, Habluetzel A, Diabate A, Sanogo-Ilboudo E, et al. (1999) Wide-scale installation of insecticide-treated curtains confers high levels of protection against malaria transmission in a hyperendemic area of Burkina Faso. Trans R Soc Trop Med Hyg 93: 473-479.

6. Aranda C, Aponte JJ, Saute F, Casimiro S, Pinto J, et al. (2005) Entomological characteristics of malaria transmission in Manhica, a rural area in southern Mozambique. J Med Entomol 42: 180-186.

7. Drakeley C, Schellenberg D, Kihonda J, Sousa CA, Arez AP, et al. (2003) An estimation of the entomological inoculation rate for Ifakara: a semi-urban area in a region of intense malaria transmission in Tanzania. Trop Med Int Health 8: 767-774.

8. Alonso PL, Lindsay SW, Armstrong JR, Conteh M, Hill AG, et al. (1991) The effect of insecticide-treated bed nets on mortality of Gambian children. Lancet 337: 1499-1502.

9. Becher H, Kynast-Wolf G, Sie A, Ndugwa R, Ramroth H, et al. (2008) Patterns of malaria: cause-specific and all-cause mortality in a malaria-endemic area of west Africa. Am J Trop Med Hyg 78: 106-113.

10. D'Alessandro U, Olaleye BO, McGuire W, Langerock P, Bennett S, et al. (1995) Mortality and morbidity from malaria in Gambian children after introduction of an impregnated bednet programme [see comments]. Lancet 345: 479-483.

11. Etard J-F, Le Hesran J-Y, Diallo A, Diallo J-P, Ndiaye J-L, et al. (2004) Childhood mortality and probable causes of death using verbal autopsy in Niakhar, Senegal, 1989-2000. International Journal of Epidemiology 33: 1286-1292(1287).

12. Trape JF, Pison G, Preziosi MP, Enel C, Desgrees du Lou A, et al. (1998) Impact of chloroquine resistance on malaria mortality. C R Acad Sci III 321: 689-697.

13. Robert V, Dieng H, Lochouran L, Traore SF, Trape JF, et al. (1998) [Malaria transmission in the rural zone of Niakhar, Senegal]. Trop Med Int Health 3: 667-677.

14. Ghana VAST Study Team (1993) Vitamin A supplementation in northern Ghana: effects on clinic attendances, hospital admissions, and child mortality. Lancet 342: 7-12.

15. Greenwood BM, David PH, Otoo-Forbes LN, Allen SJ, Alonso PL, et al. (1995) Mortality and morbidity from malaria after stopping malaria chemoprophylaxis. Trans R Soc Trop Med Hyg 89: 629-633.

16. Lindsay SW, Shenton FC, Snow RW, Greenwood BM (1989) Responses of Anopheles gambiae complex mosquitoes to the use of untreated bednets in The Gambia. Med Vet Entomol 3: 253-262.

17. Premji Z, Ndayanga P, Shiff C, Minjas J, Lubega P, et al. (1997) Community based studies on childhood mortality in a malaria holoendemic area on the Tanzanian coast. Acta Trop 63: 101-109.

18. Premji Z, Lubega P, Hamisi Y, McHopa E, Minjas J, et al. (1995) Changes in malaria associated morbidity in children using insecticide treated mosquito nets in the Bagamoyo district of coastal Tanzania. Trop Med Parasitol 46: 147-153.

19. Shiff CJ, Minjas JN, Hall T, Hunt RH, Lyimo S, et al. (1995) Malaria infection potential of anopheline mosquitoes sampled by light trapping indoors in coastal Tanzanian villages. Med Vet Entomol 9: 256-262.

20. Temu EA, Minjas JN, Coetzee M, Hunt RH, Shift CJ (1998) The role of four anopheline species (Diptera: Culicidae) in malaria transmission in coastal Tanzania. Trans R Soc Trop Med Hyg 92: 152-158.

21. Schumacher R, Swedberg E, Diallo M (2002) Mortality study in Guinea: investigating the causes of death in children under 5. Published by Save the Children Federation, Inc. and the Basic Support for Institutionalizing Child Survival (BASICS II) Project.

22. Snow RW, Mung'ala VO, Foster D, Marsh K (1994) The role of the district hospital in child survival at the Kenyan Coast. Afr J Health Sci 1: 71-75.

23. Snow RW, Bastos de Azevedo I, Lowe BS, Kabiru EW, Nevill CG, et al. (1994) Severe childhood malaria in two areas of markedly different falciparum transmission in east Africa. Acta-Trop 57: 289-300.

24. Mbogo CM, Mwangangi JM, Nzovu J, Gu W, Yan G, et al. (2003) Spatial and temporal heterogeneity of Anopheles mosquitoes and Plasmodium falciparum transmission along the Kenyan coast. Am J Trop Med Hyg 68: 734-742.

25. Mbogo CN, Snow RW, Khamala CP, Kabiru EW, Ouma JH, et al. (1995) Relationships between Plasmodium falciparum transmission by vector populations and the incidence of severe disease at nine sites on the Kenyan coast. Am J Trop Med Hyg 52: 201-206.

26. Dia I, Diallo D, Duchemin JB, Ba Y, Konate L, et al. (2005) Comparisons of human-landing catches and odor-baited entry traps for sampling malaria vectors in Senegal. J Med Entomol 42: 104-109.

27. Roca-Feltrer A, Armstrong Schellenberg JR, Smith L, Carneiro I (2009) A simple method for defining malaria seasonality. Malar J 8: 276.

28. Mapping Malaria Risk in Africa (2008) Duration of Malaria Transmission Season.
